# Principles in the Management of Glioblastoma

**DOI:** 10.3390/genes15040501

**Published:** 2024-04-17

**Authors:** Domingos Roda, Pedro Veiga, Joana Barbosa Melo, Isabel Marques Carreira, Ilda Patrícia Ribeiro

**Affiliations:** 1 Algarve Radiation Oncology Unit—Joaquim Chaves Saúde (JCS), 8000-316 Faro, Portugal; domingosmroda@gmail.com; 2 Institute of Cellular and Molecular Biology, Cytogenetics and Genomics Laboratory, Faculty of Medicine, University of Coimbra, 3000-548 Coimbra, Portugal; pedro.miguel_veiga@sapo.pt (P.V.); mmelo@fmed.uc.pt (J.B.M.);; 3 Coimbra Institute for Clinical and Biomedical Research (iCBR) and Center of Investigation on Environment Genetics and Oncobiology (CIMAGO), Faculty of Medicine, University of Coimbra, 3000-548 Coimbra, Portugal; 4 Center for Innovative Biomedicine and Biotechnology (CIBB) and Clinical Academic Center of Coimbra (CACC), University of Coimbra, 3000-548 Coimbra, Portugal

**Keywords:** genetic and epigenetic biomarkers, liquid biopsies, signaling pathways, glioblastoma, target therapies, mutations

## Abstract

Glioblastoma, the most aggressive and common malignant primary brain tumour, is characterized by infiltrative growth, abundant vascularization, and aggressive clinical evolution. Patients with glioblastoma often face poor prognoses, with a median survival of approximately 15 months. Technological progress and the subsequent improvement in understanding the pathophysiology of these tumours have not translated into significant achievements in therapies or survival outcomes for patients. Progress in molecular profiling has yielded new omics data for a more refined classification of glioblastoma. Several typical genetic and epigenetic alterations in glioblastoma include mutations in genes regulating receptor tyrosine kinase (RTK)/rat sarcoma (RAS)/phosphoinositide 3-kinase (PI3K), p53, and retinoblastoma protein (RB) signalling, as well as mutation of isocitrate dehydrogenase (*IDH*), methylation of O^6^-methylguanine-DNA methyltransferase (*MGMT*), amplification of epidermal growth factor receptor vIII, and codeletion of 1p/19q. Certain microRNAs, such as miR-10b and miR-21, have also been identified as prognostic biomarkers. Effective treatment options for glioblastoma are limited. Surgery, radiotherapy, and alkylating agent chemotherapy remain the primary pillars of treatment. Only promoter methylation of the gene *MGMT* predicts the benefit from alkylating chemotherapy with temozolomide and it guides the choice of first-line treatment in elderly patients. Several targeted strategies based on tumour-intrinsic dominant signalling pathways and antigenic tumour profiles are under investigation in clinical trials. This review explores the potential genetic and epigenetic biomarkers that could be deployed as analytical tools in the diagnosis and prognostication of glioblastoma. Recent clinical advancements in treating glioblastoma are also discussed, along with the potential of liquid biopsies to advance personalized medicine in the field of glioblastoma, highlighting the challenges and promises for the future.

## 1. Introduction

Glioblastoma originates from astrocytic glial cells [1] and is a higher-grade malignant glioma (grade 4) according to the World Health Organization (WHO) classification. This highly aggressive cancer has a poor prognosis, with a survival rate of only 15 months after diagnosis [2]. The overall 5-year relative survival rate is one of the lowest among all cancer types (4–5%), and in the last three decades, improvements in survival rates for glioblastoma patients have been very limited [2]. The current standard of care treatment includes surgical resection followed by radiotherapy and chemotherapy.

Glioblastoma is characterized by its highly invasive and therapy-resistant nature. Clinical trials testing new drugs have been on the rise, mostly those involving immunotherapy and targeted therapies [3]. Recently, glioblastoma has been classified into three subtypes based on transcriptome analysis of tumours: proneural, classical, and mesenchymal [4,5]. The mesenchymal subtype of glioblastoma is associated with the worst prognosis, highlighting the importance of these molecular data in advancing personalized treatment. However, translating the growing knowledge about glioblastoma biology into clinical practice and achieving significant improvements in therapies or patient outcomes remains challenging.

In this review, we have discussed recent advances in the management of glioblastoma, as well as the current challenges and future directions for research.

## 2. Epidemiology

Glioblastoma has a global incidence of less than 10 cases per 100,000 persons and its prevalence is even lower in the paediatric population, although this rate varies worldwide [6]. It accounts for approximately 16% of all central nervous system tumours and constitutes 54% of all gliomas [2].

Regarding age and gender, glioblastoma can occur at any age but tends to affect older adults. Its incidence increases with age, reaching a peak at 75–84 years, with the median age at diagnosis being 64 years old [2]. Glioblastoma is more common in males than in females [2,6]. In terms of race and ethnicity, there is a higher incidence of glioblastoma and poorest survival in Caucasians when compared to Asians and Black individuals [6,7]. While most glioblastomas occur without any apparent family history, there are certain genetic conditions that have been associated with an increased risk of developing primary brain tumours, such as Li–Fraumeni, Turcot, BRCA syndrome and neurofibromatosis type 1, among others [8].

Exogenous factors may also play a role in the origin of glioblastoma and other primary central nervous system tumours. Currently, several studies are attempting to establish an association between the development and progression of the disease and environmental factors, such as exposure to radiation and toxic elements, dietary habits, lifestyle, mobile phone use, or exposure to electromagnetic waves [9,10,11]. Certain studies have provided evidence that exposure to radiation and toxic agents, such as pesticides, can increase susceptibility to the development of this disease [9,12]. The correlation between disease development and factors such as lifestyle, mobile phone use and dietary habits remains inconclusive [13,14,15].

## 3. Classification of Glioblastoma

The fourth WHO classification of gliomas from 2016 is based on the degree of malignancy, as determined by histopathological criteria, in which four types of this neoplasm have been distinguished [16]:

− Glioblastoma, isocitrate dehydrogenase (IDH) wildtype (90% of cases), developing *de novo* at about 60 years of age;
− Glioblastoma, IDH-mutant (10% of cases), secondary glioblastoma that usually develops in younger patients with gliomas of higher differentiation (WHO grades I–III); it carries a significantly better prognosis than wildtype IDH [17];
− Glioblastoma not otherwise specified (NOS), the IDH mutation status could not be determined due to a lack of histological or molecular material for testing;
− Not-elsewhere-classified (NEC) glioblastoma, fourth category distinguished in recent years.

For NEC, the necessary decision to classify the tumour had been made, but the results did not allow assignment of the tumour to any of the above categories of the 2016 WHO classification. This may occur if there are differences among the clinical, histological, immunohistological and genetic characteristics of the tumour. It is also possible that there are unknown combinations of characteristics of glioblastoma subunits that have not yet been classified by the WHO division [6,16]. Their consecutive classifications are strongly related to histological features and clinical outcome, together with the relatively recent incorporation of molecular characteristics. The 5th edition of the World Health Organization (WHO) classification of tumours of the central nervous system (CNS), published at the end of 2021, has introduced new taxonomy and nomenclature of a great number of tumours including gliomas [18].

During the last decade, there has been a paradigm shift in CNS tumour diagnostics as advances in molecular genetics have revealed alterations in these tumours. In the 2016 WHO classification, molecular changes have been introduced into the diagnostic workup of some tumours and a comprehensive multi-layered diagnosis has been established that includes histopathological and molecular information [16,19]. The WHO 2021 version (WHO CNS5) considers molecular genetics and clinical relevance to a greater extent, so the latest version includes elements of both histopathology and molecular genetics, resulting in a somewhat hybrid taxonomy [20,21]. In this setting, the classifications include three genetic variables (*TERT* promoter mutation, *EGFR* gene amplification, +7/−10 chromosome copy number variations) as criteria to diagnose glioblastoma (grade 4), IDH-wildtype [20,22].

In this classification, to make the diagnosis of a glioblastoma the following are required [20]:

- adult patient
- diffuse astrocytic tumour
- IDH-wildtype
- and at least one of the following:
  ○ necrosis
  ○ microvascular proliferation
  ○ *TERT* promoter mutation
  ○ *EGFR* gene amplification
  ○ combined gain of whole chromosome 7 and loss of chromosome 10 [+7/−10].

*MGMT* (O^6^-methylguanine-DNA methyltransferase) promoter methylation status is an essential part of molecular diagnostics for all high-grade gliomas (grade 3 and 4). *MGMT* promoter methylation is associated with better survival outcomes in patients with high-grade glioma and is a predictive factor for response to treatment with alkylating chemotherapy, as pointed out by major clinical guidelines in association with the classification [22].

## 4. Molecular Pathogenesis of Glioblastoma

### 4.1. Cell Signalling Pathways in Glioblastoma

The molecular pathogenesis of glioblastoma is complex and involves multiple alterations in various genes, cell signalling pathways, and genome regulatory elements. Several candidate genes in glioblastoma have been described that may influence the development and progression of the disease—*CDKN2A*, *TP53*, *EGFR*, *PTEN*, *NF1*, *CDK4*, *RB1*, *IDH1*, *PIK3CA*, and *PIK3R1*. These genes are implicated in various cell signalling pathways such as the PI3K/AKT/mTOR pathway, RAS/RAF/MAPK pathway, and p16INK4a/CDK4/Rb pathway [23].

The PI3K/AKT/mTOR pathway involves receptor tyrosine kinases (RTKs) such as EGFR and a tumour suppressor protein, PTEN, which acts as an antagonist of PI3K, contributing to the downregulation of this pathway and inhibiting cell proliferation. In the absence of PTEN, PI3K phosphorylates phosphatidylinositol-4,5-bisphosphate (PIP2) into phosphatidylinositol-3,4,5-trisphosphate (PIP3), leading to the activation of the AKT protein. Both AKT and mammalian targets of rapamycin (mTOR) are serine/threonine-specific protein kinases (STKs) that play key roles in cell proliferation. This pathway is dysregulated in 80% of glioblastomas [24].

**Figure 1:**
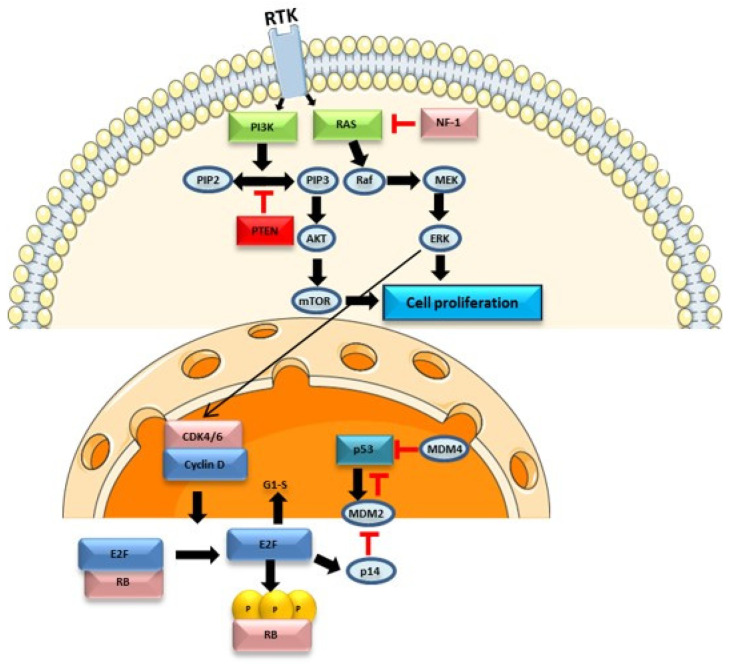
Simplified schematic representation of the PI3K/AKT/mTOR, RAS/RAF/MAPK, RB and p53 pathways and how they interact. The activation of the PI3K/AKT/mTOR and RAS/RAF/MAPK pathways by the binding of growth factors to the RTKs plays an important role in cell proliferation. MDM2 and MDM4 are negative regulators of p53. p14 inhibits MDM2, contributing to p53 expression. These pathways interact in different ways, highlighting potential targets of therapeutic intervention. Parts of the figure were drawn using Servier Medical Art licensed under a Creative Commons Attribution 3.0 Unported License.

The RAS/RAF/MAPK pathway (Figure 1) is involved in the regulation of apoptosis, proliferation, cell differentiation, and development. In this signalling pathway, multiple isoforms of RAS (H-RAS, N-RAS, K-RAS) and RAF play distinct roles and exhibit oncogenic potential. RAS activation is controlled by binding to guanosine triphosphate (GTP) and its inhibition occurs by binding to guanosine diphosphate (GDP). RAS activation leads to RAF kinase activation, regulating downstream signalling pathways such as the mitogen-activated protein kinase (MAPK) pathway [25,26]. The BRAF protein is a serine/threonine kinase belonging to the RAF family, whose alteration is also associated with brain tumours. The BRAFV600E mutation is particularly relevant, leading to the activation of the RAS/RAF/MAPK pathway, promoting cell proliferation and survival, and inhibiting apoptosis. However, this missense alteration is rare in glioblastoma, detected in only 1–2% of cases [27,28]. This signalling pathway also interacts with other pathways, namely the PI3K/AKT/mTOR pathway and the p53 protein, contributing to the normal functioning of cell processes. Activation of this pathway can also lead to activation of hypoxia-inducible factor-1α (HIF-1α) and vascular endothelial growth factor receptor (VEGF), promoting tumorigenesis and angiogenesis [29]. Dysregulation of this pathway may contribute to increased cell proliferation, as seen in various glioblastoma cases with RAS overexpression. Additionally, growth factors such as EGFR, PDGFR, and other RTKs are often overexpressed, further contributing to glioblastoma pathogenesis. In this context, this signalling pathway may constitute a potential therapeutic target [25].

The Rb protein, encoded by the *RB1* gene (retinoblastoma 1), controls the cell cycle between the transition of the G1 to S phase. In normal cells, cyclin D1 activates CDK4, phosphorylating the Rb protein, releasing the E2F1 transcription factor, and contributing to cell cycle progression (Figure 1). CDK4 activity is suppressed by INK4 family proteins. Approximately 78% of glioblastoma show alterations in the p16INK4a/CDK-4/Rb signalling pathway [24]. The RB1 gene is typically downregulated in glioblastoma, contributing to cell cycle progression.

Cell cycle checkpoints are crucial for activating DNA repair pathways in case of DNA damage. In certain cases, cyclin-dependent kinase inhibitors may be activated by the p53 protein, encoded by the TP53 gene, blocking the cell cycle and activating apoptosis pathways, preventing tumour development. However, 87% of glioblastoma patients have mutations in the TP53 gene, an important tumour suppressor gene [23,24].

Cell signalling pathways are interconnected, acting in different cellular processes. Therefore, alterations in genes related to one pathway may have implications in others, contributing to the complexity of glioblastoma pathogenesis [29]. Currently, there is also evidence that certain cell pathways are regulated by epigenetic mechanisms, which may contribute to disease progression and therapy resistance [30].

### 4.2. Epigenetic Mechanisms

Epigenetic modifications, such as DNA methylation, histone modifications, chromatin remodelling, and non-coding RNAs, play a significant role in the development and progression of cancer [30]. The analysis of epigenetic modifications in glioblastoma allows for better stratification of patients and can serve as therapeutic targets [31]. Promoter methylation of the MGMT gene, an important repair gene, appears to be associated with a favourable prognosis and a better response to temozolomide (TMZ) treatment [32].

Another relevant mechanism is histone modification, where changes in epigenetic regulatory genes, such as HDACs (histone deacetylases), histone demethylases and methyltransferases contribute to glioblastoma. Targeting histone acetylation as a therapeutic strategy has gained attention in the field of glioblastoma research. Histone deacetylase (HDAC) inhibitors have been investigated as potential treatments. By inhibiting HDACs, these drugs aim to increase histone acetylation and promote a more open chromatin structure, potentially reactivating tumour suppressor genes and inhibiting oncogenes [30].

Chromatin remodelling is closely associated with histone modifications. Genes involved in chromatin remodelling are frequently mutated or dysregulated in glioblastomas [33]. Ganguly et al., 2018, demonstrated that the protein BRG1, the catalytic subunit of the SWI/SNF chromatin remodelling complex, contributes to maintaining glioma-initiating cells (GlCs) in their stem-like state, promoting the development of the tumour and contributing to their heterogeneity. Furthermore, inhibition of BRG1 sensitized GlCs to chemotherapy and TMZ, suggesting that this protein could be a novel therapeutic target in glioblastoma [34]. However, chromatin remodelling complexes are complicated structures that still require more knowledge of their regulatory mechanisms and their implications in the genesis of glioblastoma.

**Figure 2:**
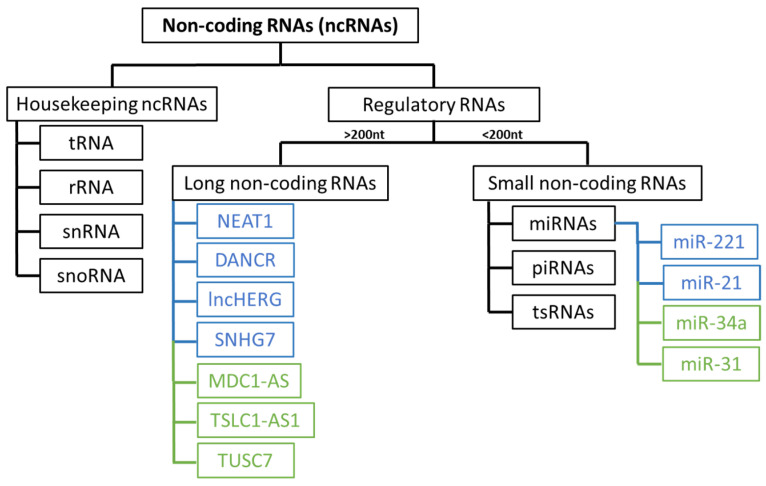
Non-coding RNAs (ncRNAs) classification and some lncRNAs and miRNAS with oncogenic (blue) and tumor suppressor (green) potential in glioblastoma. Housekeeping ncRNAs include transfer RNA (tRNA), ribosomal RNA (rRNA), small nuclear RNA (snRNA) and small nucleolar RNA (snoRNA). Regulatory RNAs include long non-coding RNAs and small non-coding RNAs. MicroRNAs (miRNAs), PIWI-interacting RNAs (piRNAs), and tRNA-derived small RNAs (tsRNAs) are small non-coding RNAs.

Recently, there has been a growing focus on understanding the role of non-coding RNAs (ncRNAs) in the development and progression of glioblastoma. These RNA molecules are divided into several groups (Figure 2) and play an important role in regulating gene expression [35,36].

Dysregulation of microRNAs is common in various types of cancer, including glioblastoma [37]. The loss of tumour suppressor miRNAs, which typically regulate mRNAs that may have oncogenic potential, such as miR-31, which inhibits CDKN2A/B and miR-34a, affecting EGFR protein levels, contributes to cell proliferation, apoptosis resistance, and subsequent disease progression [36]. The overexpression of other miRNAs, such as miR-221 and miR-21 (oncomiRs), facilitates cell growth and proliferation in glioblastoma by interacting with various genes involved in key signalling pathways [37].

Long non-coding RNAs (lncRNAs) are frequently dysregulated in various types of cancers, including glioblastoma, and this dysregulation can alter the activity of oncogene promoters, facilitating interactions with transcription factors and contributing to cell proliferation and disease progression [35,36,38]. Numerous oncogenic lncRNAs associated with glioblastoma, such as NEAT1, DANCR, lncHERG, SNHG7, MNX1-AS1, MCM3AP-AS1, and LINC01446, contribute to invasion, migration, chemoresistance, apoptosis resistance, and cell proliferation [39]. Certain lncRNAs have tumour suppressor functions and are typically downregulated, such as MDC1-AS, TSLC1-AS1, ADAMTS9-AS2, and TUSC7 [40].

From a therapeutic point of view, epigenetic modifications can be associated with treatment resistance and can contribute to tumour development. Therefore, targeting epigenetic regulators as a therapeutic strategy can offer advantages for the clinical management of the disease [38]. Moreover, understanding the interactions between the cell signalling pathways involved in glioblastoma’s pathogenesis and epigenetics offers promising insights into potential therapeutic targets and personalized treatments, providing better outcomes for the patients.

### 4.3. Multi-Omics Approach

An approach that involves the integration of genomics, transcriptomics, proteomics, and other omics data allows researchers to gain a comprehensive understanding of the underlying molecular mechanisms in glioblastoma development. This integration of diverse omics data facilitates the identification of genetic variants, gene expression changes, and protein alterations, contributing to the stratification of patients based on molecular alterations present in the tumour. Additionally, it potentially allows for the discovery of novel therapeutic targets [41]. With the technological progress in the molecular characterization of cancer, we may be a step closer to glioblastoma personalized medicine, offering individualized diagnosis and targeted therapies, which will contribute to better management of the disease.

## 5. Prognostic Biomarkers

**Figure 3:**
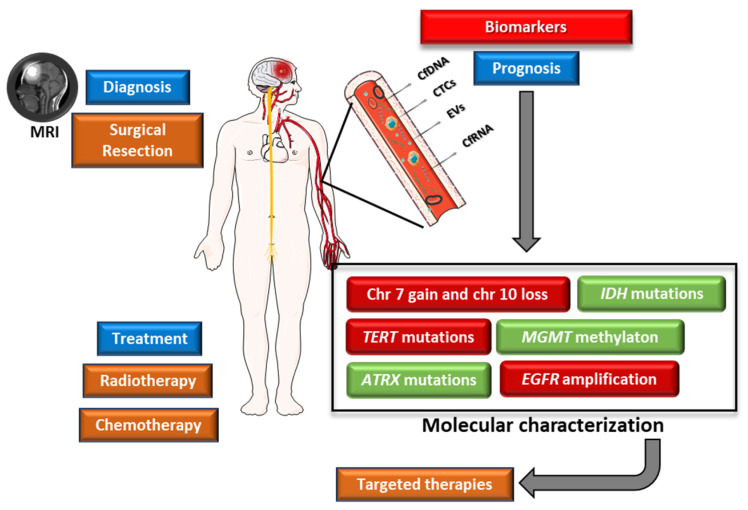
Glioblastoma management overview. Glioblastoma diagnosis is based on MRI and biopsy samples. Current standard treatment consists of maximal surgical resection followed by radiation and adjuvant TMZ. Liquid biopsy emerges as a promising tool in the management of this disease, ultimately contributing to the development of targeted therapies. Parts of the figure were drawn using Servier Medical Art licensed under a Creative Commons Attribution 3.0 Unported License.

The invasive nature of glioblastoma is a critical factor in its aggressive growth and progression. It is therefore essential to identify recurring cytogenetic, genomic, and epigenomic changes that contribute to the development of this cancer. These changes have the potential to serve as genetic biomarkers, offering insights to improve patient care and treatment outcomes and therefore may influence the survival rates of this highly aggressive disease (Figure 3) [32]. Several genetic alterations are used as potential prognostic biomarkers in glioblastoma (Table 1).

### 5.1. IDH1 and IDH2 Mutational Status

Isocitrate dehydrogenase 1 (IDH1) and isocitrate dehydrogenase 2 (IDH2) are enzymes that play crucial roles in cell metabolism, particularly in the Krebs cycle. IDH1 is primarily located in the cytoplasm and peroxisomes of cells. Its main function is to catalyse the oxidative decarboxylation of isocitrate to produce α-ketoglutarate [42]. This enzyme also plays a role in maintaining the balance of NADP+ and NADPH, protecting cells against oxidative stress. IDH2 has a similar function as IDH1 [43].

Mutations in *IDH1* or *IDH2* are positive prognostic factors, improving the prognosis of the patients when compared to IDH-wildtype glioblastomas. *IDH* mutations lead to a gain-of-function mutation where the enzyme produces the oncometabolite D-2-hydroxyglutarate (D2HG) instead of the normal product, α-ketoglutarate (αKG) [44]. This metabolite inhibits αKG-dependent dioxygenases that are involved in the regulation of epigenetics and differentiation, which is thought to induce epigenetic dysfunction and therefore slows down tumour growth by inhibiting normal cell differentiation. Elevated levels of D2HG are also reported to induce DNA hypermethylation which may reduce tumour cell proliferation since hypermethylation is associated with gene silencing [32].

### 5.2. Alterations in ATRX (ATRX Chromatin Remodeler)

The *ATRX* gene provides instructions for making a protein involved in gene regulation by a process known as chromatin remodelling. *ATRX* is frequently mutated in astrocytomas IDH-mutant. In terms of prognosis, in patients diagnosed with glioblastoma IDH-wildtype, *ATRX* alterations were associated with favourable outcomes [32].

### 5.3. Alterations in TERT (Telomerase Reverse Transcriptase)

The *TERT* gene encodes a subunit of telomerase, which is responsible for maintaining telomeres. Mutations in the promoter of this gene are associated with an increase in telomerase activity, contributing to cell immortalization [45]. Arita et al., 2013 concluded that in 98% of the tumours analysed, a mutation in the promoter of the *TERT* gene was present concomitantly with 1p/19q loss and IDH1/2 mutations [46]. Mutations in the *TERT* gene are associated with a worse prognosis, particularly in IDH-wildtype gliomas [32].

### 5.4. Alterations in CDKN2A (Cyclin Dependent Kinase Inhibitor 2A)

The *CDKN2A* gene encodes a protein of the INK4 family, which acts as a cell cycle inhibitor, controlling cell cycle progression. The main function of this protein is to inhibit the action of cyclin-dependent kinases 4 and 6 (CDK4/6) by preventing the phosphorylation of retinoblastoma, blocking the entry of cells to the S phase [47]. Homozygous deletion of this gene is associated with a poor prognosis [32].

### 5.5. 1p/19q Codeletion

The 1p/19q codeletion represents a translocation t(1;19)(q10;p10), and in addition to its prognostic significance, it is also utilized for the classification of the tumour [20,32]. Regarding its value as a prognostic biomarker, this alteration is associated with a favourable prognosis and chemosensitivity in lower-grade gliomas [32]. Clark et al., state that the 1p/19q codeletion has no prognostic impact on gliomas classified as glioblastomas [48]. In addition, this alteration is uncommon in glioblastomas. However, Mizoguchi et al., studied a small group of glioblastoma patients with 1p/19q deletion who had a more favourable clinical outcome [49].

Data regarding the impact of this alteration in glioblastoma remains limited, and thus, further studies are necessary to understand the significance of this alteration in the management of this disease.

### 5.6. Chromosome 7 Gain and Chromosome 10 Loss

Gain of chromosome 7 and loss of chromosome 10 are common alterations in glioblastoma and are typically associated with a poor prognosis [32]. These alterations can involve the entire chromosome or be partial and are considered a molecular marker of IDH-wildtype glioblastomas [50]. Chromosome 10 monosomy is associated with the loss of an important tumour suppressor gene—*PTEN* (phosphatase and tensin homolog). This gene encodes a phosphatase protein that plays a crucial role in regulating cell growth and division by interacting with phosphatidylinositol 3,4,5-triphosphate (PIP3), thus blocking the PI3K/AKT pathway and inhibiting cell proliferation [51].

On the other hand, trisomy of chromosome 7 is associated with the amplification of oncogenes, particularly the *EGFR* (epidermal growth factor receptor) gene, contributing to increased cell proliferation and disease progression [32].

### 5.7. EGFR Mutations

The *EGFR* gene encodes for a transmembrane receptor with tyrosine kinase activity involved in various cell signalling pathways, such as PI3K/AKT/mTOR and RAS/RAF/MAPK, participating in multiple cellular processes, including cell proliferation, apoptosis, differentiation, cell growth, and migration [52]. Overexpression of this growth factor due to mutations or amplifications of this gene is often detected in glioblastoma [32,52]. Most patients with *EGFR* amplification have a deletion of exons 2–7 (EGFRvIII). This variant is usually expressed by extrachromosomal DNA fragments called “double minutes” [53].

Amplification of this gene is associated with a poor prognosis as the tumour has an increased capacity for proliferation, cell migration, neovascularization, and resistance to chemotherapy [52,54]. However, despite numerous studies reporting this negative association [32,55,56,57], Ohgaki et al., 2004 reported that in a cohort of 715 glioblastoma patients, the presence of *EGFR* gene amplification did not affect overall survival [58]. Faulkner et al., 2015 also concluded that overexpression of this gene is not a predictive biomarker of overall survival based on data from a cohort of 51 individuals, 49% of whom had alterations in the *EGFR* gene [59]. Therefore, there is a need for further studies to better understand the impact of this alteration on the management of this disease.

In addition to its function as a potential predictive biomarker, *EGFR* gene amplification is also used for glioblastoma classification [20].

### 5.8. MGMT Promoter Methylation

*MGMT* (O^6^-methylguanine-DNA methyltransferase) encodes a protein involved in DNA repair, specifically responsible for removing alkyl groups from the O^6^ position of guanine. Temozolomide is an alkylating agent that induces DNA damage by adding a methyl group to the N^7^ and O^6^ positions of guanine and the N^3^ position of adenines. This alteration leads to activation of the MMR (mismatch repair) pathway during DNA replication, culminating in double-strand breaks and ultimately leading to apoptosis [60]. The MGMT protein prevents this by removing and transferring the methyl group, thus inhibiting the cytotoxic action of TMZ. Consequently, promoter methylation of the *MGMT* gene is associated with a favourable prognosis and a better response to TMZ treatment [32]. Several studies confirmed this association and reported a higher overall survival and better response to treatment compared to patients without methylation of the *MGMT* gene [61,62,63,64,65]. For patients in this situation, O^6^-benzylguanine (O^6^-BG) can be used for MGMT inactivation. This O^6^-meG analogue passes through the blood–brain barrier so it can be used as a potential treatment for glioblastoma, sensitizing cells to TMZ [66].

**Table 1:** Diagnostic, prognostic and predictive biomarkers in glioblastoma.

Biomarker	Classification	Effect	References
*IDH1* and *IDH2*	Prognostic	Mutations in *IDH1* or *IDH2* are positive prognostic factors	[44]
*ATRX*	Prognostic	*ATRX* alterations are associated with a better prognosis	[32]
*TERT*	Diagnostic and prognostic	Mutations in the *TERT* gene are associated with a worse prognosis	[32]
*CDKN2A*	Prognostic	Homozygous deletion of this gene is associated with a poor prognosis	[32]
1p/19q Codeletion	Prognostic	1p/19q deletion is associated with a more favourable clinical outcome, although this alteration is uncommon in glioblastoma	[49]
7+/10−	Diagnostic and prognostic	Gain of chromosome 7 and loss of chromosome 10 are common alterations in glioblastoma and are typically associated with a poor prognosis	[32]
*EGFR*	Diagnostic, prognostic and predictive	Amplification of this gene is associated with a poor prognosis and resistance to therapy	[52,54]
*MGMT*	Prognostic and predictive	Promoter methylation of the *MGMT* gene is associated with a favourable prognosis and a better response to treatment	[32]

It is important to acknowledge that even though some of these biomarkers have a positive impact on the patient’s prognosis, they are just one aspect of the complex molecular and genetic landscape of glioblastoma.

### 5.9. Liquid Biopsy

Liquid biopsies allow the detection of nucleic acids (DNA or RNA), circulating tumour cells (CTCs), and extracellular vesicles (EVs), from blood samples, cerebrospinal fluid (CSF), urine, or other body fluid. In the context of glioblastoma, the most relevant are CSF [67], blood [68], and, although with limited evidence, urine [69]. Currently, glioblastoma diagnosis relies on imaging and histopathological analysis, requiring surgical intervention for tissue sampling and subsequent molecular analysis [32].

The principal benefit of this non-invasive approach lies in its capacity for early detection of the disease, tracking the possibility of relapse after treatment, and providing a means for molecular tumour profiling through a simple blood or other body fluid sample. Liquid biopsy also allows the patient to be followed throughout treatment, without the need for multiple surgical interventions during the course of the disease.

Glioblastoma is characterized by high heterogeneity, leading to diverse cell populations with various molecular alterations. Therefore, a tissue biopsy may not provide an accurate representation of the tumour [70]. The main objective of liquid biopsies is to detect biomarkers with implications for disease management, treatment, prognosis, and monitoring [71]. Despite advances, liquid biopsies also have limitations, particularly in tumour representation, as some alterations may remain undetected due to the low concentrations of relevant biomarkers in biological fluids. Therefore, larger cohort studies are needed to determine the sensitivity and effectiveness of liquid biopsies in glioblastoma molecular characterization.

Cerebrospinal fluid is in close contact with the brain and spinal cord, making it a receptor for various cell-secreted products. Also, it is not separated from the tumour by the blood–brain barrier (BBB) so it may have a greater representation of the tumour cells than plasma samples. Compared to blood samples, CSF is obtained through an invasive method, making it challenging to repeat at key timepoints during patient follow-up. Several studies [67,72,73,74,75] demonstrated the possibility of detecting ctDNA in CSF, making it a strong candidate for its use in liquid biopsies, with the potential to significantly impact tumour characterization, diagnosis, prognosis, and disease management [67]. Pan et al., 2019 showed that the molecular profile obtained by sequencing ctDNA from CSF coincided with the tumour profile and, in some cases, ctDNA analysis detected genetic alterations that had not been found in the tumour [74]. This can be explained due to the limitations in the collection of the tissue and tumour heterogeneity.

The analysis of biomarkers present in plasma has been studied in the oncological context, namely in neurological tumours, as it allows their molecular study and patient follow-up without the use of multiple surgical interventions [71]. In contrast to CSF, blood is subject to the BBB. Therefore, for a liquid biopsy using a plasma sample to be representative of the tumour, the materials secreted by tumour cells must be able to cross this barrier [76]. The integrity of the BBB changes during the development of a brain tumour, becoming more permeable and forming a brain–tumour barrier (BTB). This alteration is attributed to the downregulation of tight junctions, primarily due to the overexpression of vascular endothelial growth factor (VEGF), a mitogen associated with angiogenesis [77]. Thus, the overexpression of this growth factor can constitute a prognostic biomarker and be used as a target for the development of targeted therapies [78]. While the BBB presents a challenge for liquid biopsies, numerous studies have demonstrated potential for the detection of circulating tumour DNA (ctDNA) in blood samples. Some of these studies report detection rates ranging from 27% to 55% using next-generation sequencing (NGS) techniques [79,80,81]. This detection rate is consistent with findings by Bettegowda et al., 2014, where ctDNA was detected in less than 50% of patients with primary brain tumours, compared to 75% of patients with other types of cancers [82]. However, some of these studies have limitations regarding the number of patients. Although the use of plasma as a biofluid for liquid biopsies seems promising, it is essential to consider that the molecular profile of the tumour may be underrepresented, because the molecular alterations in tissue may not entirely reflect those detected in plasma due to the BBB. So, the results obtained should be interpreted cautiously and integrated with data obtained from complementary methods.

Regarding the use of urine samples in liquid biopsies for molecular characterization of glioblastoma, there are relatively few conclusive studies. Some report that it is possible to detect ctDNA in urine but have some limitations in the size of the cohort, which does not allow the clinical impact to be demonstrated [69]. Moreover, it is important to note that urine is produced by the kidneys, and numerous components might not be as effectively filtered into urine compared to blood. Consequently, urine-based liquid biopsy has received more extensive attention in genitourinary cancers, while it poses a significant challenge in non-urological cancers [83].

#### 5.9.1. Circulating Tumour Cells

Circulating tumour cells (CTCs), first described by Ashworth in 1869 [84], constitute a heterogeneous group of cells originating from the primary tumour or metastatic sites, which dissociate from the tumour and enter the bloodstream as either individual cells or clusters [85,86]. This type of cell contributes to metastasis and its detection can serve as a predictive biomarker and a way of monitoring treatment response [76]. However, the proportion of CTCs in the bloodstream is quite low, especially in the early stages of the disease, posing significant challenges to their isolation and characterization. Consequently, an initial enrichment step is necessary for CTC isolation. Currently, for most tumours, this selection process is based on the expression of the epithelial cell adhesion molecule (EpCAM). Nonetheless, glioblastoma tumour cells exhibit a mesenchymal phenotype and, consequently, lack EpCAM expression [76]. Thus, new methods are needed to isolate this type of cell. The detection of CTCs in cases of glioblastoma is also challenging, as this type of cancer rarely has extracranial metastases, largely due to the low survival rate and the constitution of central nervous system tissues [87]. Several studies demonstrated the possibility of detecting CTCs using different isolation methods, including density-gradient centrifugation, immunomagnetic enrichment, Parsortix microfluidic cassettes, and size-based techniques [88,89,90,91]. Sullivan et al. detected CTCs in 39% of the patients enrolled in their study through a blood-based liquid biopsy. They also concluded that patients in an advanced state of the disease exhibited a higher frequency of CTCs, further emphasizing the utility of CTC analysis in disease monitoring [92]. Despite these challenges, the study of CTCs allows for a more comprehensive molecular analysis, allowing characterization at the DNA, RNA, and protein levels. Therefore, CTCs can serve as a valuable predictive and prognostic biomarker. However, more evidence of their impact on the management of the disease is needed before applying this technology in clinical practice [32]. Furthermore, to date, there are few studies dedicated to CTC analysis in glioblastoma, with most of them enrolling a limited number of patients [88,91,92].

#### 5.9.2. Cell-Free Nucleic Acids

Liquid biopsy also allows the detection of nucleic acids (DNA/RNA), whose main objective is the detection of point mutations, copy number variations (CNVs) in specific genes, or methylation status, which may have a significant impact on diagnosis, prognosis, and treatment [93]. ctDNA is a component of cell-free DNA (cfDNA) that is released by the tumour into the bloodstream and other body fluids due to cell death and apoptosis. It comprises DNA fragments of approximately 180–200 base pairs and sequencing of these fragments allows the detection of a wide spectrum of changes that may have an impact on the disease [32]. The analysis of ctDNA from CSF appears to have a higher sensitivity, representing the tumour molecular profile in a more reliable way [71]. Regarding the use of blood samples, plasma should be used in ctDNA analysis, since compared to serum it has lower levels of background cfDNA resulting from the cell lysis of lymphocytes [94]. However, ctDNA levels in plasma are also variable among patients, depending on the disease progression, tumour heterogeneity, effects of the treatment and individual patient characteristics. In most cases, low levels of ctDNA are associated with the presence of the BBB [93,94]. Several studies used different techniques for ctDNA detection and analysis, including NGS [95] and PCR-based methods such as digital droplet PCR (ddPCR) [96]. These methods allow the detection of various genetic alterations commonly found in glioblastoma, such as mutations in the *EGFR*, *PTEN*, *TP53*, *TERT,* and *RB1* genes [96,97]. Muralidharan et al., 2021 developed a novel ddPCR assay for the detection of two mutations in the promotor of the *TERT* gene from cfDNA. They obtained a sensitivity of 62.5% and a specificity of 90% compared to detection in a tissue sample [98]. Through sequencing, Mouliere et al., 2021 detected ctDNA from plasma, CSF, and urine samples of patients with gliomas, demonstrating high sensitivity. Despite the limited number of individuals included, they demonstrated the promising use of liquid biopsies in the management of the disease [69]. However, there is a need for more translational studies to establish liquid biopsy as a routine clinical practice in the management of glioblastoma. In addition to point mutations and CNVs, the analysis of ctDNA also allows the study of methylation. In a study conducted by Dai et al., 2023, a genome-wide analysis of methylation was performed on ctDNA extracted from CSF, demonstrating the possibility of integrating epigenetic studies into the management of this disease [99]. In glioblastoma, the analysis of *MGMT* methylation patterns significantly impacts prognosis and treatment response.

RNA analysis can also serve as a biomarker for monitoring disease progression and response to treatment. Like cfDNA, cfRNA can also be extracted from CSF, blood, and urine [100]. cfRNA originates from tumour cells and is released into the bloodstream through necrotic or apoptotic cells or a vesicle-free RNA-binding protein-dependent pathway. There are several types of cfRNA, with microRNAs and lncRNAs being the most studied in the context of liquid biopsy [52]. Some microRNAs act as tumour suppressors, including miR-7, miR-34a, miR-128, miR-181a, and miR-181b, which are typically downregulated in glioblastoma. Upregulated microRNAs (oncomiRs) influence the expression of tumour suppressor genes and promote oncogenesis [101]. The most studied oncomiRs in glioblastoma are miR-21, miR-10b, miR-93, miR-221, miR-222, and miR-182 [101,102]. LncRNAs interact with miRNAs, contributing to the regulation of diverse signalling pathways, including NOTCH (neurogenic locus notch homolog protein), MAPKs (mitogen-activated protein kinases), PI3K/AKT/mTOR (phosphoinositide 3-kinase/protein kinase B/mammalian target of rapamycin), Wnt/β-catenin, and BMP (bone morphogenetic protein). Dysregulation of these cell pathways is associated with the development and progression of glioblastoma, and therefore, lncRNAs have potential as predictive biomarkers since they can also influence response to treatment [103].

Wu et al., 2020 reported that LINC00470 promoted cell proliferation, invasion, and resistance to TMZ by competitively interacting with miRNA-134, which targets MYC, negatively affecting its expression [104].

#### 5.9.3. Extracellular Vesicles

In the context of liquid biopsies, extracellular vesicles (EVs) have gained greater focus in recent studies, although they were first reported in 1946 by Chargaff and West [105]. These vesicles are small structures enclosed by a lipid membrane and are secreted by cells. EVs can originate from the endosomal system and are referred to as exosomes or be released from the plasma membrane, known as microvesicles [106,107]. The main interest in the isolation of these structures lies in the informative power they carry since they can transport nucleic acids, lipids, and proteins, acting as vehicles of intercellular communication [103]. EVs can be isolated from blood, CSF, urine, or other body fluids. The use of EVs in liquid biopsies seems promising since they are more stable than CTCs and cfDNA/RNA and are able to pass the BBB, even when it is intact [108].

Tumour cells also release these vesicles, which can contribute to metastasis, angiogenesis, and chemotherapy resistance by affecting other cells within the tumour microenvironment and more distant cells or organs [107]. One of the main challenges in the application of EVs analysis in liquid biopsies is related to the difficulty in isolating these structures that are susceptible to contamination with non-EV proteins, lipoproteins, and high-density lipoproteins (HDL). Currently, there are several methods for the isolation of EVs that can be divided based on their physical and chemical properties. These methods may include centrifugation-based isolation, size, affinity, precipitation, or microfluidic techniques [107,108]. Ma et al., 2022 demonstrated that EVs are released by glioma stem cells and alter the tumour microenvironment, making tumour cells resistant to treatment. However, these results are only based on data obtained from cell lines from three patients [109]. More recently, Tzaridis et al., 2023 investigated differences in the quantity of EVs in the serum of 67 patients with glioblastoma and 22 controls. They found a significant increase in EVs in the patient group, highlighting the possibility of isolating these structures from a blood liquid biopsy. Nevertheless, the authors also reinforce the limitations regarding the number of patients and heterogeneity within the cohort itself, which does not allow definitive conclusions to be drawn [110]. On the other hand, Garcia et al., 2019, concluded that exosome concentrations were higher in patients compared to controls and also demonstrated the possibility of isolating exosomes from plasma samples. This study also had a limited number of participants (19 patients and 19 controls), emphasizing the inherent limitations that are common to several studies [111].

While the use of EVs as predictive biomarkers in glioblastoma appears promising, further research involving larger cohorts is needed to obtain more data concerning clinical applicability and disease management. Standardization of laboratory practices in EVs isolation is also required to overcome methodological challenges.

## 6. Diagnosis

**Figure 4:**
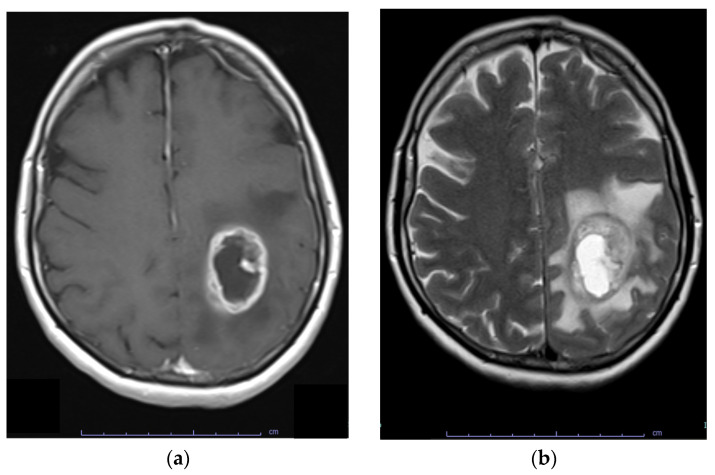
Contrast-enhanced MRI of a glioblastoma. (**a**) Contrast-enhanced T1 image.; (**b**) T2 image showing oedema.

Most glioblastoma are diagnosed after the onset of symptoms because they rapidly expand or infiltrate brain structures. Indicative symptoms may include new-onset seizures, progressive headache, focal neurological symptoms, mental status changes, and signs of increased intracranial pressure [112,113]. Contrast-enhanced MRI is the diagnostic tool of choice for glioblastoma. These lesions are infiltrative and diverse, originating and spreading from the white matter. Involvement of the corpus callosum is frequently observed. Glioblastomas are poorly circumscribed and display contrast enhancement at their margin as a sign of blood–brain barrier disruption. The core of the abnormal tissue shows decreased signal intensity on T1-weighted imaging as a result of necrosis. Enhancement (gadolinium) is variable but is almost always present, typically peripheral and irregular with nodular components, and it usually surrounds the necrosis. Commonly, the surrounding area exhibits increased signal intensity on T2-weighted and fluid-attenuated inversion recovery (FLAIR) images, indicating cerebral oedema. (Figure 4). Multicentric enhancement, haemorrhage, and cystic changes are also frequent [114].

## 7. Current Treatment Options

### 7.1. Management of Newly Diagnosed Glioblastoma

Radical microsurgical resection of a glioblastoma is limited by the highly invasive nature of the tumour, with infiltrating tumour cells typically extending significant distances from the main tumour mass [115]. Nonetheless, the goal of glioblastoma surgery should be gross total resection of the enhancing solid tumour mass whenever feasible. Although some studies reported progressive improvements in outcomes as the extent of resection increased beyond 78%, in both newly diagnosed [116,117] and recurrent glioblastoma, only gross total resection was associated with improved outcomes [118,119].

The extent of resection (EOR) is a highly studied subject in relation to the surgical management of glioblastoma. Firstly, it has been demonstrated that EOR has an impact on the overall survival (OS) of patients with glioblastoma. However, there has been considerable discussion on the ideal threshold for EOR. The paper by Lacroix et al. reported that an EOR > 98% or higher resulted in a significant improvement in median survival (8.8 months vs. 13 months, *p* < 0.0001) [120].

Shah et al. investigated the impact of supramaximal resection or anatomic lobectomy on the survival rates of patients diagnosed with non-eloquent gliomas. A propensity-matched analysis demonstrated that supramaximal resection led to enhanced overall survival (30.7 vs. 14.1 months) and progression-free survival (17.2 vs. 8.1 months) in comparison to the gross total resection group (*p* < 0.001) [121].

With the intent to achieve more control of the quality of the resection, 5-aminolevulinic acid (5-ALA) was introduced in the surgery setting. 5-ALA is a natural precursor of hemoglobin and is a fluorescent dye that is preferably picked up by tumour cells after being orally administered 2–3 h prior to surgery. 5-ALA-guided surgery is highly effective in accurately detecting malignant tumour tissues during surgery. It is an intraoperative tool that may be used independently of neuronavigation to achieve the maximum extent of tumour removal without causing neurological impairment [122,123].

Following maximal safe resection, adjuvant involved-field radiotherapy (RT) with concurrent (75 mg/m^2^/day × 6 wk.) and adjuvant (150–200 mg/m^2^/day × 5 days for six 28 day-day cycles wk.) temozolomide (TMZ) is the standard treatment recommended for patients with newly diagnosed glioblastoma based on the results of the phase III, randomized EORTC-NCIC study [124].

In this study, 573 patients, with newly diagnosed glioblastoma who were aged ≤70 years and had a WHO PS ≤2 were assigned to treatment. Overall survival was 27.2% (95% CI 22.2–32.5) at 2 years, 16.0% (12.0–20.6) at 3 years, 12.1% (8.5–16.4) at 4 years, and 9.8% (6.4–14.0) at 5 years with temozolomide, versus 10.9% (7.6–14.8), 4.4% (2.4–7.2), 3.0% (1.4–5.7), and 1.9% (0.6–4.4) with radiotherapy alone (hazard ratio 0.6, 95% CI 0.5–0.7; *p* < 0.0001) [22,124].

The new guidelines recommend this approach with or without alternating electric field therapy (TTFields) [22]. Even though the spectrum of effects elicited remains incompletely understood, emerging data suggest that TTFields exert biophysical forces on a variety of charged and polarisable molecules to elicit a multitude of biological processes with anticancer effects, including DNA repair, autophagy, cell migration, permeability, and immunological responses. Substantial geographic variation in TTFields availability exists in the clinical practice, following cost-effectiveness continuous evaluations [125].

Even in the elderly, radiation therapy has confirmed a modest improvement in survival without a detriment in quality of life or neurocognition [126].

Patients who are elderly (≥70 years), have reduced performance status, significant medical comorbidities, or significant neurologic deficits can be treated with a diversity of established hypofractionated (higher dose per fraction over fewer total treatments) schedules ranging from 5 to 15 fractions [127,128].

### 7.2. Radiotherapy Considerations

For resected tumours, GTV delineation should be based on the resection cavity (if present) and any residual enhancing tumour on contrast-enhanced T1-weighted MRI, regardless of peritumoral oedema [129].

**Figure 5:**
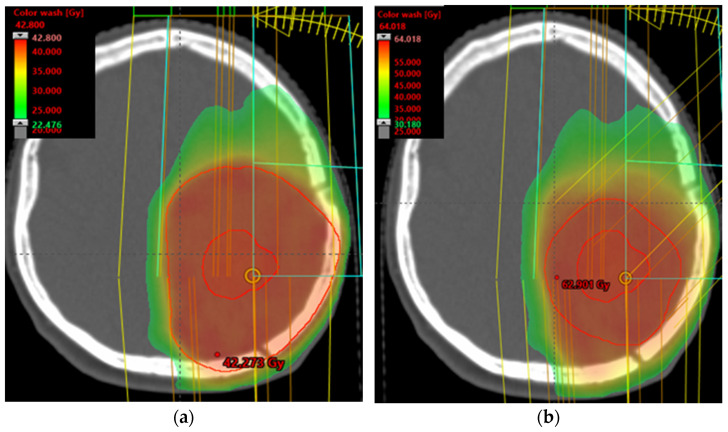
(**a**) Isodose distribution first course to 40 Gy intensity-modulated radiation therapy (IMRT)/volumetric modulated arc therapy (VMAT) radiation technique; (**b**) isodose distribution boost dose to 60 Gy (IMRT)/(VMAT) radiation technique.

Although the European Organization for Research and Treatment of Cancer (EORTC) and the Radiotherapy and Oncology Group (RTOG) have adopted different methods for delineating target volumes in glioblastoma, both groups have formerly recommended a volumetric GTV expansion of 2 cm to generate the CTV. This margin was applied to embrace areas of potential microscopic tumour infiltration and was adjusted to respect anatomical borders, as described in other preceding glioblastoma target delineation guidelines [129,130] (Figure 5).

Recent, retrospective, and prospective studies, applying reduced GTV-to-CTV margins of 0.5–1.5 cm with either conventionally fractionated or hypofractionated radiation schedules, have shown that overall survival, progression-free survival times, and recurrence patterns are similar to those found in guidelines using current target delineation recommendations [128,131].

Proton beam therapy (PBT) enables high-dose irradiation of tumours without increasing the dose to the surrounding normal tissue by applying a sharp energy peak called the Bragg peak, which has an acute distal dose fall-off in its depth-dose distribution protecting normal surrounding tissue [132]. Recent authors demonstrated that high-dose PBT (96.6 Gy in 56 fractions by hyperfractionated concomitant boost) conferred a statistically significant survival advantage in patients with glioblastoma compared to conventional radiation therapy using propensity-matched cohorts. Although acute radiation-related toxicities were equivalent between the PBT and CRT groups, radiation necrosis as a late radiation-related toxicity was more prevalent in the PBT group [133]. The possibility of access to this radiotherapy technique is still scarce in many places around the world.

## 8. Novel Treatment Options

### 8.1. Targeted Therapies: From Cell Signalling Pathways to Cell Metabolism

Targeted therapies aim to interfere with the function of a specific molecule involved in the genesis of glioblastoma, affecting the cascade of reactions downstream of the pathway where this molecule acts. Despite advances in understanding the molecular mechanisms and molecular profile of glioblastoma, there has not yet been significant progress with applicability in clinical practice [27,118].

Several clinical trials (Table 2) targeted different cell signalling pathways related to the development and progression of glioblastoma.

Some of these trials use drugs that target receptor tyrosine kinases (RTKs) or their respective cell pathways, specifically directed at *EGFR, MET, FGFR, BRAF* mutations, neurotrophic tyrosine receptor kinases (NTRK), or the PI3K/AKT/mTOR pathway. Others target elements of cell cycle regulation and apoptosis pathways, such as RB, the p53 pathway, and TERT. Drugs acting on elements of the tumour microenvironment, such as VEGF and transforming growth factor-β (TGF-β), are also being investigated [27,134]. Targeted therapies may include inhibitors of the target molecule, vaccines, CAR T-cells, or antibodies [134].

Regarding clinical trials targeting proteins in the PI3K/AKT/mTOR pathway, specifically NCT01339052 and NCT01019434, buparlisib demonstrated minimal single-agent efficacy in patients with PI3K-activated recurrent glioblastoma, and temsirolimus was not superior to temozolomide in patients with an unmethylated *MGMT* promoter, respectively [135,136]. However, Wen et al., concluded that buparlisib achieved significant brain penetration, but the inhibition of the signalling pathway in the tumour tissue was incomplete, which can explain the low efficacy [135].

In the cases of certain drugs specifically targeting receptor tyrosine kinases (RTKs) in clinical trials (NCT01268566; NCT01632228; NCT01975701), the results were not promising. Despite good tolerance, their use as monotherapy or in combination with others demonstrated limited efficacy, showing a lack of clinical benefit [137,138,139].

More recently, a clinical trial (NCT04121455) showed good tolerance and safety regarding the therapeutic approach. This trial included several patients who underwent biopsy or incomplete resection of the tumour and lacked MGMT promoter hypermethylation. The combined use of radiotherapy with olaptesed pegol (NOX-A12), a CXCL12 inhibitor, demonstrated a median overall survival (OS) of 19.9 months, indicating a survival benefit for the patients [140].

Another clinical trial (NCT06102525), currently in development based on gene therapy, used an RNA-replacement enzyme that targets hTERT mRNA and replaces it with therapeutic gene RNA, contributing to the modulation of the tumour microenvironment by reducing VEGF expression, thus making it more responsive to immunotherapy.

Wick et al. randomly assigned patients with progression after chemoradiation in a 2:1 ratio to receive lomustine plus bevacizumab or lomustine alone. The combination therapy did not provide a survival advantage; the median overall survival was 9.1 months in the combination group and 8.6 months in the monotherapy group (hazard ratio for death, 0.95; 95% CI, 0.74 to 1.21; *p* = 0.65). PFS survival was 2.7 months longer in the combination group than in the monotherapy group: 4.2 months versus 1.5 months (hazard ratio for disease progression or death, 0.49; 95% CI, 0.39 to 0.61; *p* < 0.001 [141].

The BELOB trial was an open-label, three-group, phase 2 study. Adult patients with a first recurrence of a glioblastoma after temozolomide chemoradiotherapy were randomly allocated to treatment with oral lomustine 110 mg/m^2^ once every 6 weeks, intravenous bevacizumab 10 mg/kg once every 2 weeks, or combination treatment with lomustine 110 mg/m^2^ every 6 weeks and bevacizumab 10 mg/kg every 2 weeks. The 9-month overall survival was 43% in the lomustine group, 38% in the bevacizumab group, and 63% (49–75) for the combined bevacizumab and lomustine groups. These results permit the use of this combination in off-label use in some centres [142].

Lee et al. performed a systematic review on the risks and benefits of the use of anti-EGFR therapies in glioblastomas. It was observed there was no evidence of a benefit in OS with the use of anti-EGFR therapy in the first-line or recurrent setting (hazard ratio (HR) 0.89, 95% confidence interval (CI) 0.76 to 1.04). The addition of anti-EGFR therapy showed no evidence of an increase in progression-free survival (PFS) in the first-line setting. In the recurrent setting, there was an increase in PFS with the use of anti-EGFR therapy [143].

Tumour metabolism can also serve as a therapeutic target, as it may be involved in the development and progression of glioblastoma. Tumour cells show increased glycolysis, known as the Warburg effect, providing a proliferative advantage in hypoxic conditions. Alterations in other metabolic processes such as oxidative phosphorylation, the pentose phosphate pathway (PPP), lipids, amino acids, and nucleotides metabolism have also been described [144,145].

Some ongoing clinical trials related to tumour metabolism (Table 1) are still in early phases and, therefore, do not yet have available results. Clinical trials NCT03032484 and NCT02029573, despite both treatments being well-tolerated and safe, lack evidence of their effectiveness in patient survival [146,147].

Despite advances in understanding the molecular pathogenesis of glioblastoma and its impact on the development of targeted therapies, further studies should be carried out to expand the knowledge of the relationship between cell metabolism, cell signalling pathways, the tumour microenvironment, and genomic alterations. This will allow the development of new targeted therapies with a significant impact on the management of the disease.

### 8.2. Immunotherapies

Immunotherapy aims to use the patient’s immune system to target cancer cells. This approach has shown promising results in various types of cancer, particularly in hematological cancers. Immunotherapy may include checkpoint inhibitors, chimeric antigen receptor (CAR) T-cells, monoclonal antibodies, vaccines, and immune system modulators [148]. Regarding glioblastoma, few advances have been made and it remains in the clinical trial phase.

The brain is considered an immune-privileged organ due to the presence of the BBB, which limits the access of immune system cells and poses a challenge to the development of targeted therapies. Glioblastoma also develops an immunosuppressive microenvironment, which stimulates disease progression. Glioblastoma cells release chemokines, growth factors, and cytokines, which attract and stimulate cells with immunosuppressive function [148,149]. Glioblastoma cells also exhibit overexpression of programmed death receptor ligand 1 (PD-L1), which interacts with the receptor PD-1 on T cells, inhibiting their function and leading to a decreased immune response [149]. Hypoxia within the tumour microenvironment has been associated with increased expression of hypoxia-inducible factor 1-α (HIF1-α). Overexpression of HIF1-α can compromise T cell activity and upregulate immunomodulators such as transforming growth factor β (TGF-β) and vascular endothelial growth factor (VEGF), promoting immunosuppression [148,150].

The intricate and distinctive tumour microenvironment of glioblastoma may contribute to the reduced efficacy of immunotherapy. In this context, there are several clinical trials to determine the efficacy of immunotherapy, some of which are described in Table 3.

In the clinical trial NCT03726515, using CART-EGFRvIII cells, any clinical benefit could not be established. However, the authors concluded that these intravenously infused cells did reach the tumour and exert antigen-directed activity, although the tumour microenvironment became even more immunosuppressive after treatment with the CAR T cells [151]. In the clinical trial NCT01109095, treating progressive glioblastoma with HER2-CAR VSTs was found to be feasible and safe, resulting in clinical benefit for 8 out of 17 patients. In this cohort, the median overall survival was 24.5 months from diagnosis [152]. The combination of CAR T cells with checkpoint inhibition (NCT04003649) is also under study in the context of glioblastoma.

Vaccines have also been explored in glioblastoma treatment. The use of rindopepimut with temozolomide for patients with newly diagnosed, EGFRvIII-expressing glioblastoma did not demonstrate any survival benefit in a phase III trial [153]. On the other hand, a phase II trial (NCT01498328) reported favourable results in a cohort of 73 patients with recurrent EGFRvIII-positive glioblastoma [78].

The use of monoclonal antibodies such as avelumab (NCT03047473) and nivolumab (NCT02017717), belonging to the immune-checkpoint inhibitor class, failed to demonstrate efficacy for checkpoint blockade, bringing no improvement in overall survival [78,154]. On the contrary, the use of atezolizumab combined with TMZ and radiotherapy (NCT03174197) showed modest efficacy [155].

Weiss et al., 2021, combined the use of TMZ with L19TNF in newly diagnosed glioblastoma patients (NCT04443010). So far, this clinical trial is still in the recruitment phase and has not yet shown results. L19TNF represents a fully human antibody–cytokine fusion protein, composed of TNF (tumour necrosis factor) fused to the L19 antibody, which binds to a tumour-specific epitope of the extracellular matrix protein fibronectin (EDB-FN). This allows the precise delivery of therapeutic TNF doses exclusively to the tumour, without affecting other regions [156].

Although immunotherapy does not yet have clinical applicability in the case of glioblastoma, the various clinical trials may provide important information for the development of more robust clinical trials with larger sample sizes. These trials should consider addressing issues such as antigen escape, tumour heterogeneity, the tumour microenvironment, drug delivery strategies, patient selection based on tumour genetics, and multimodal approaches [148]. Furthermore, clinical trials with unmet endpoints contribute to a better understanding of the role of the tumour microenvironment in immune responses, leading to the development of new strategies and drugs with different molecular targets or the combination of different therapeutic approaches, improving outcomes and disease management.

### 8.3. Obstacles in Using Targeted Therapies

Despite numerous studies with several promising in vitro results regarding glioblastoma, there is still difficulty in implementing targeted therapies that are effective. Several challenges can affect the efficacy of these treatments, namely tumour heterogeneity, BBB, tumour microenvironment, redundant cell signalling pathways and the presence of glioblastoma stem cells (GSCs) [27,118].

The presence of diverse cell clones with different molecular profiles makes it challenging to employ targeted therapies for specific alterations or signalling pathways. Thus, tumour heterogeneity may account for the failures observed in in vivo clinical trials based on monotherapy [27].

The BBB is an obstacle to drug delivery to the brain, preventing the passage of 98% of all small molecules. While this barrier may be compromised in certain regions due to tumour development, other locations, typically the infiltrative tumour edge left behind after resection, may maintain its integrity preventing the passage of the therapeutic agent [27,118].

Redundant pathways share the same downstream signalling targets and, therefore, can contribute to increased therapy resistance [118]. The tumour microenvironment, characterized by hypoxia, plays a role in angiogenesis, promoting migration, invasion, and chemoresistance.

Glioblastoma stem cells (GSCs) arise from the malignant transformation of neural stem cells or through dedifferentiation of tumour cells following radiation or chemotherapy. These cells exhibit high proliferative capacity, metastatic potential, and the ability to suppress the anti-inflammatory response, contributing to a greater resistance to treatment [27].

All of these factors can contribute to the acquisition of resistance to therapies, affecting their effectiveness.

## 9. Conclusions

Glioblastoma is a highly malignant, and aggressive, primary brain tumour that causes substantial neurological impairment. The current standard of care comprises the optimal surgical removal of the tumour, simultaneous administration of chemotherapy and radiation therapy, and subsequent use of adjuvant chemotherapy.

Despite major advancements in comprehending the molecular pathophysiology and biology of glioblastoma, there has been limited translation of this knowledge into substantial improvements in patient outcomes. Molecular and genetic biomarker testing will become a regular part of the eligibility requirements for clinical studies. These studies are necessary to evaluate the ability of substances to cross the blood–brain barrier and their effects on the body’s response to drugs. Additionally, incorporating imaging and a wide range of biomarkers will help identify patients who are more likely to benefit from treatment based on the unique biology of their tumours.

The challenges in glioblastoma clinical trial design stem from inherent factors related to these tumours, including the limited pool of eligible patients and the subsequent challenges in conducting large randomized trials. Another challenge lies in comprehensively understanding the extensive genetic and molecular heterogeneity observed among different glioblastomas.

The assessment of response or ‘biological activity’ will involve the measurement of biomarkers at the molecular level, in addition to the use of established clinical and imaging criteria.

By incorporating a broader range of medical disciplines, spanning from laboratory research to patient care, it is expected that this heightened awareness will result in the discovery of more efficient and refined treatments.

## Figures and Tables

**Table 2:** Examples of clinical trials using targeted therapies in glioblastoma.

	Trial	Molecular Target	Therapy	Status
Cell signalling pathways	NCT01339052	PI3K	Buparlisib	Completed (phase II)
	NCT00943826	VEGF	Bevacizumab + TMZ	Completed (phase III)
	NCT01019434	mTOR	Temsirolimus + RT	Completed (phase II)
	NCT05222802	EGFR	ERAS-801	Recruiting (phase I)
	NCT02981940	CDK4/6	Abemaciclib	Active, not recruiting (phase II)
	NCT01268566	PDGFR	MEDI-575	Completed (phase II)
	NCT01632228	MET	Onartuzumab	Completed (phase II)
	NCT05376800	MDM2	Brigimadlin	Recruiting (phase 0/Ia)
	NCT01975701	FGFR	BGJ398	Completed (phase II)
	NCT02340156	p53	SGT-53 + TMZ	Terminated (phase II)
	NCT02345824	CDK4/6	Ribociclib	Unknown status (phase I)
	NCT04121455	CXCL12	NOX-A12 + RT	Active, not recruiting (phase I/II)
	NCT06102525	hTERT	RZ-001 + Valganciclovir	Not yet recruiting (phase I/IIa)
	NCT01582269	TGF-β	Galunisertib	Active, not recruiting (phase II)
Tumour cell metabolism	NCT04825275	HK2—glucose metabolism	Posaconazole	Recruiting (phase 0)
	NCT04587830	Arginine metabolism	ADI-PEG 20 + RT + TMZ	Recruiting (phase Ib)
	NCT03032484	FASN—lipid metabolism	TVB-2640 + Bevacizumab	Completed (phase II)
	NCT04869449	HK2—glucose metabolism	Ketoconazole	Recruiting (early phase I)
	NCT02029573	HMGCR—cholesterol Metabolism	Atorvastatin + RT + TMZ	Completed (phase II)

**Table 3:** Examples of clinical trials using immunotherapy in glioblastoma.

Trial	Molecular Target	Type of Therapy	Status
NCT03726515	EGFRvIII	CAR T + Pembrolizumab	Completed (phase I)
NCT00895180	PDGFR	Monoclonal antibody	Completed (phase II)
NCT01454596	EGFRvIII	CAR T	Completed (phase I/II)
NCT00128635	DNA–histone H1 complex	Monoclonal antibody	All completed (phase I and phase II the NCT00004017)
NCT00004017			
NCT00509301			
NCT01498328	EGFRvIII	Vaccine	Completed (phase II)
NCT01109095	HER2	CAR T	Completed (phase I)
NCT04214392	CLTX	CAR T	Recruiting (phase I)
NCT03174197	PD-1/PD-L1	Monoclonal antibody with a checkpoint inhibitor function + RT + TMZ	Active, not recruiting (phase I/II)
NCT04443010	EDB-FN	Cytokines + TMZ	Recruiting (phase I/II)
NCT03047473	PD-1/PD-L1	Monoclonal antibody with a checkpoint inhibitor function	Completed (phase II)
NCT02049489	CD133	Vaccine	Completed (phase I)
NCT05024175	EGFRvIII	CAR-T	Not yet recruiting (phase I)
NCT04047706	PD-1/PD-L1	Monoclonal antibody with a checkpoint inhibitor function	Active, not recruiting (phase I)
NCT02017717	PD-1/PD-L1	Monoclonal antibody with a checkpoint inhibitor function	Active, not recruiting (phase III)
NCT04661384	IL13Rα2	CAR T	Recruiting (phase I)
NCT01480479	EGFRvIII	Vaccine + TMZ	Completed (phase III)
NCT04003649	IL13Rα2	CAR T with checkpoint inhibition	Recruiting (phase I)

TMZ—Temozolomide; CAR T—Chimeric antigen receptor T-cell.

## Data Availability

Not applicable.
